# Experimental host preference of diapause and non-diapause induced *Culex pipiens pipiens* (Diptera: Culicidae)

**DOI:** 10.1186/s13071-015-1012-1

**Published:** 2015-07-24

**Authors:** Ary Faraji, Randy Gaugler

**Affiliations:** Center for Vector Biology, Department of Entomology, Rutgers University, New Brunswick, New Jersey 08901-8536 USA; Salt Lake City Mosquito Abatement District, 2020 North Redwood Road, Salt Lake City, Utah 84116-1248 USA

**Keywords:** Host selection, Avian feeding, Mammalian feeding, Overwintering, Bird-baited traps

## Abstract

**Background:**

*Culex pipiens pipiens* plays an important role in the transmission of several vector-borne pathogens such as West Nile virus (WNV) in North America. Laboratory and field studies suggest that this species is ornithophilic but because of genetic hybridization with sibling species during the active mosquito season, it may occasionally feed on mammals. Adult female *Cx. p. pipiens* undergo a facultative diapause and may serve as an overwintering mechanism for WNV. To determine the effect of diapause on the innate host preference of *Cx. p. pipiens* emerging from winter hibernation, we conducted host-choice experiments using bird and mammal hosts.

**Methods:**

Mosquitoes were reared under non-diapause induced (NDI), diapause induced (DI), and field collected from overwintering (OW) hibernaculae. They were released into a large mesh enclosure housing two lard can traps, and given a choice between feeding on a dove or a rat.

**Results:**

Host seeking *Cx. p. pipiens* were four times more likely to feed on the dove than the rat, regardless of experimental conditions. Under NDI conditions, *Cx. p. pipiens* were (*p* < 0.001) more attracted to the bird (79.9 % [75.6-84.1]) than the rat (20.1 [15.9-24.4]). Overwintering mosquitoes and those exposed to DI conditions were also significantly (*p* < 0.001) more attracted to birds (81.6 % [75.9-87.3]) than to rats (18.5 [12.7-24.2]).

**Conclusions:**

We provide new information about the innate host preference of *Cx. p. pipiens* emerging from diapause in temperate habitats where winter survival is crucial for disease transmission cycles. Although we showed that *Cx. p. pipiens* prefers an avian to a mammalian host, nearly 20 % of emerging mosquitoes in the spring could feed on mammals. Changes in host preferences may also contain valuable clues about transmission dynamics and subsequent timely interventions by vector control and public health practitioners.

## Background

West Nile virus (WNV) and St. Louis encephalitis (SLE) are serious public and veterinary health concerns. These viruses are primarily maintained in an enzootic cycle involving ornithophilic *Culex* mosquitoes and avian amplification hosts [1–3]. Although mammals succumb to arboviral infections, they do not develop prolonged or high levels of viremia and are considered dead-end or dilution hosts [4]. Increased mammalian feeding by the primary vectors will negatively affect disease ecology because amplification of these avian zoonoses depend on virus consistently reaching competent hosts.

*Culex pipiens pipiens* L. has been incriminated as the primary enzootic vector of WNV and SLE throughout its geographic range [1, 2, 5, 6]. This mosquito belongs to a complex of evolutionarily and morphologically closely related species which differ dramatically in biology, ecology, and vectorial capacity [2, 7]. In northeastern USA, *Cx. p. pipiens* populations are composed of two forms: *Cx. p. pipiens* form “pipiens” and *Cx. p. pipiens* form “molestus” [2, 8]. *Culex p. pipiens* form pipiens develops in above ground larval habitats, mates in swarms within open areas, undergoes a facultative winter diapause, requires a blood meal to develop eggs, and primarily feeds on birds [2, 7, 9]. Conversely, *Cx. p. pipiens* form molestus develops in underground larval habitats, mates in confined spaces, remains active throughout winter in sequestered subterranean habitats, can produce the first batch of eggs autogenously, and prefers to feed on mammals [2, 9]. The pipiens and molestus forms are generally reproductively isolated in nature, but occasionally hybridize, producing females that may feed indiscriminately on avian or mammalian hosts [6, 8–13]. Thus, the primary enzootic vector may also serve as an epizootic or epidemic bridge vector in ecological cycles of mosquito-borne pathogens in northeastern USA [6, 12].

Field investigations in northeastern USA have shown that *Cx. p. pipiens* derive 93-96 % of their blood meals from avian sources [14–17]. However, additional field evidence from the mid-Atlantic and upper Midwestern USA have shown that some populations may derive 13-22 % of their blood meals from mammalian sources [12, 18]. However, higher fractions of molestus ancestry have been detected in *Cx. p. pipiens* populations with mammalian-derived blood meals [10, 13]. It has been hypothesized that genetic ancestry may drive *Cx. p. pipiens* to occasionally feed on mammals, but the rates of vector-host contact may also be influenced by other factors. These include the innate host preference of the vector, host availability, spatial or temporal variations, and even the method of sampling for host feeding determinations [19]. But an overlooked factor is the effect of diapause on the host preference of emerging populations of *Cx. p. pipiens*.

Diapause is the primary mechanism for survival in temperate environments. Diapause is a genetically determined response by which an insect enters a dormant state in response to environmental cues which indicate the onset of unfavourable conditions [20]. The dynamic state of winter diapause in *Cx. p. pipiens* is induced primarily by decreasing photoperiodic signals in the late summer and fall, and is often enhanced by cooling temperatures [7, 21–23]. Females emerging from pupae under these conditions do not blood feed but instead sequester fat bodies from carbohydrate sources, mate with males and store spermatozoa in spermatheca, seek shelter inside a hibernacula, and enter into a state of reproductive (ovarian) diapause [24–27]. The ovarian follicles of diapausing *Cx. p. pipiens* are in a state of arrest that are easily distinguishable from their summer resting stage through follicular morphometric examinations ([22, 25]. A small number of females may also become infected with arboviruses, such as WNV, through vertical (or transgenerational) transmission and may serve as a winter reservoir for the virus [28–34].

For WNV and SLE, diapausing *Cx. p. pipiens* mosquitoes are critical for harboring the viruses during winter and subsequently reintroducing them as disease agents the following spring [25, 28, 29, 35]. Diapause also regulates the size and synchronization of mosquito populations the following season. In northeastern USA, *Cx. p. pipiens* mosquitoes emerge from hibernation in April-May [22, 25, 36] and are considered critical for initiating WNV transmission among birds in the early spring [1, 37]. However, although extrinsic factors such as temperature and rainfall affect the intensity of WNV transmission [3], it is unclear why WNV levels remain consistently low until mid-summer, even after suitable temperatures for viral replication have been reached [38, 39]. We hypothesize that a reason for the weakened early season amplification of WNV may be that *Cx. p. pipiens* emerging from diapause may be feeding more frequently on mammals, which are incompetent hosts, versus the more competent avian amplification hosts. This may dampen the intensity of WNV transmission in most host communities in the early season and may explain the reduced levels of WNV detected in the spring.

We examined the attractiveness of non-diapause (NDI) and diapause induced (DI) *Cx. p. pipiens* mosquitoes to a dove or a rat via a series of host-choice experiments. We established colonies of *Cx. p. pipiens* collected as egg rafts and exposed them to simulated NDI or DI conditions and then determined the host selection of adults under laboratory conditions. We also collected overwintering (OW) *Cx. p. pipiens* from field hibernaculae, terminated ovarian diapause, and exposed the mosquitoes to the same host-choice experiments. Our primary question was to determine if *Cx. p. pipiens* emerging from winter diapause will feed indiscriminately on avian or mammalian hosts.

## Methods

### Mosquitoes

*Culex p. pipiens* mosquitoes used in our host selection trials were reared from field-collected egg rafts in Trenton, New Jersey, USA (40° 14’ 23.62” N, 74° 44’ 26.98” W). Local egg raft collections and laboratory rearing would ensure that adequate numbers of mosquitoes of the same physiological status and age were available for all experiments. We used black gravid trap pans (20 x 38 × 12.7 cm, BioQuip, Rancho Dominguez, CA, USA) filled with a fermented mixture of grass clippings and de-chlorinated tap water [40]. Egg rafts were collected in the morning and transported to the laboratory. Individual egg rafts were allowed to hatch in separate larval rearing trays containing 2 L of de-chlorinated tap water. Larvae from the same egg batch were reared in separate 20 cm × 30 cm stainless steel pans, fed finely ground rat chow on alternate days, and trays were skimmed daily to remove exuviae and debris following standard protocols [41]. All larvae were reared in incubators to ensure consistent temperature, humidity, and photoperiod regimes. Species identification was conducted on fourth instars and confirmed on adult specimens using standard keys [42].

Non-diapause induced larvae were reared under 27 °C, 70 % RH, and 16:8 h (L:D) photoperiod. Emerging adults were transferred to screened cages (30.5 × 30.5 × 30.5 cm, BioQuip, Rancho Dominguez, CA, USA), provided a 10 % sucrose solution, and maintained under the same conditions as above. Female *Cx. p. pipiens* used in the host selection trials were five days post-emergence and deprived of sucrose solution 12 h prior to testing. Five females were retained from each replicate for ovarian dissections to record follicular morphometrics of ovarioles and confirm physiological status.

Diapause induced larvae were reared under 10 °C, 70 % RH, and a photoperiod of 8:16 h to ensure induction of diapause [22, 25, 43]. Emerging adults were transferred to screened cages, provided a 10 % sucrose solution, and maintained under the same conditions as above for four weeks. Five female *Cx. p. pipiens* were retained from each batch for dissections to confirm ovarian diapause induction. Diapause was then terminated by exposing the mosquitoes to NDI conditions (27 °C, 70 % RH, 16:8 h photophase) for one week. Five additional adult females were again retained for dissections to confirm diapause termination. Subsequent adult female *Cx. p. pipiens* were then deprived of sucrose solution for 12 h prior to each host selection trial.

Field collections of overwintering *Cx. p. pipiens* were conducted within the abandoned underground ammunition bunkers of Fort Mott, Pennsville, New Jersey, USA (39° 36’ 14.85” N, 75° 33’ 13.47” W). Mosquitoes were collected on six different occasions between November 2010 to March 2011. Female mosquitoes were gently aspirated and placed in coolers with ice packs for transport back to the laboratory. Five females were retained from each collection for dissections to confirm ovarian diapause status. In the laboratory, mosquitoes were placed in screened cages, provided a 10 % sucrose solution, and exposed to NDI conditions (27 °C, 70 % RH, 16:8 h photophase) for one week to terminate diapause. Five adult females were retained for dissections to confirm diapause termination. Subsequent *Cx. p. pipiens* females were then deprived of the sucrose solution for 12 h prior to each host selection trial.

### Dissection methods

Dissections were conducted under a stereomicroscope with dissecting needles using the ovariolar separation technique of Hoc and Schaub [44]. Ovaries were teased apart to expose individual follicles for examination and measurement under 400X phase contrast microscopy [28]. Five ovarioles from each female were randomly selected and the developmental stage of the primary follicle was recorded using Christophers and Mer stages [45]. In addition, the length of the primary and secondary follicles was measured for five ovarioles of each female using an ocular micrometer, and a mean value was recorded. Measurements were used to calculate the ratio of the primary to secondary follicle for determination of ovarian diapause induction [24]. Ovarian diapause in *Cx. p. pipiens* is characterized by primary follicles which measure ≤50 μm (≥70 μm in NDI mosquitoes) and exhibit a primary to secondary follicular ratio of ≤1.6 (≥2.0 in NDI mosquitoes) [24, 25].

### Animals and cages

We tested white rock dove birds, *Columbia livia* Gmelin, and white rats, *Rattus norvegicus* (Berkenhout), of similar body mass (ca. 300 g) for all host selection trials. Animals were weighed prior to each trial and rotated with each subsequent experiment so that the same animal was not used for consecutive trials. We followed guidelines set forth in the Guide for the Care and Use of Laboratory Animals [46] as approved by the Animal Use Committee of Rutgers University under protocol No. 86–129.

**Fig. 1:**
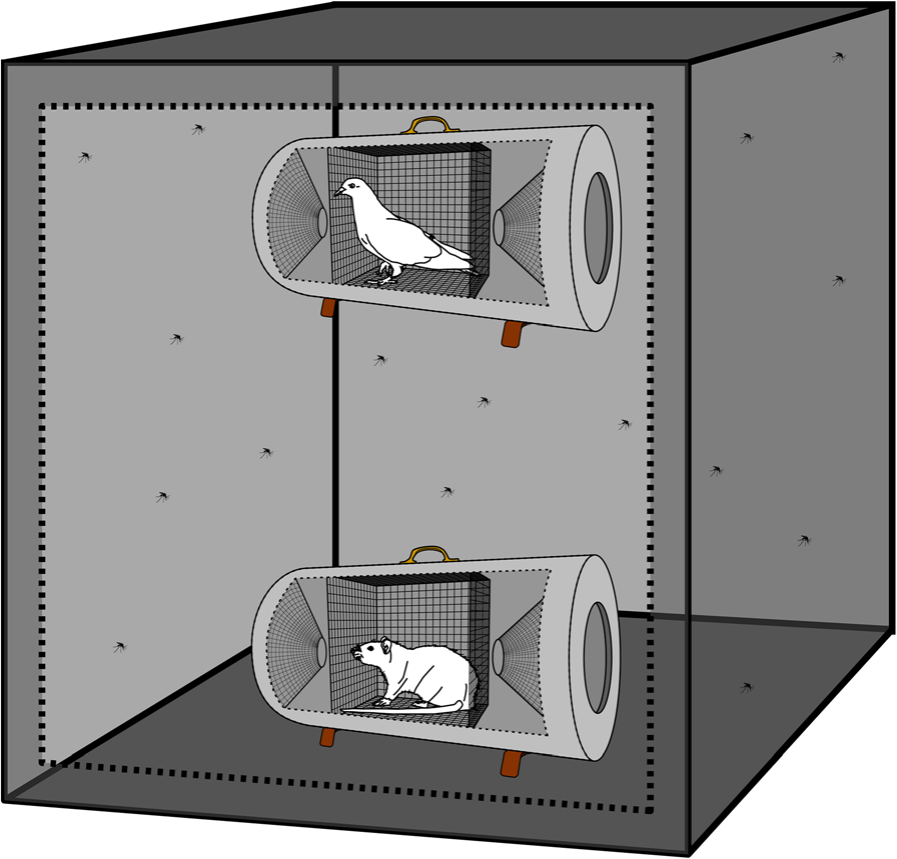
Large cage mesh enclosure and lard can animal traps used during experimental host-choice studies. The large mesh cage measured 3 x 3 x 3 m and the animal traps measured 25 x 25 x 70 cm

A single animal was placed inside a cylindrical lard can trap (25 × 25 × 70 cm) [47–49] with 2-cm mesh screening to confine the animal but allow for host odour emanation (Fig. 1). Mosquitoes entered the trap through one of two removable funnels on either end of the trap. The funnels contained a mosquito-proof mesh that prevented direct contact between the animals and mosquitoes. Lard can traps were placed inside a larger mesh enclosure (3 x 3 ×3 m) within the laboratory (Fig. 1). Entry to the enclosure for placement of the lard can traps was gained by a 1.5 × 2.5 m door and an additional 25 × 25 cm window lined with stretchable cotton cloth sleeve material allowed for insertion of mosquitoes during trials. The enclosure was lined with mosquito-proof mesh and further covered with clear vinyl plastic to retain humidity.

### Host-choice experiments

We conducted three primary host-choice experiments using NDI, DI, and overwintering mosquitoes. We released 25–70 *Cx. p. pipiens* females from the same egg batch or collection for each trial, depending on the number of available specimens. Each experiment was repeated four times to allow for placement of the bird or rat in the up and down positions. Experiments consisted of a total of 48 replicates using 1,589 mosquitoes (NDI), 36 replicates with 701 mosquitoes (DI), and 40 replicates with 2,285 mosquitoes (overwintering). Each trial was conducted 1 h before sunset to 1 h post sunrise under 27 °C, 70 % RH, and 16:8 h photophase. During each experiment, a rock dove was placed in a lard can trap hung from a small hook from the inside ceiling of the mesh enclosure (Bird Up position) and a rat was placed in another lard can trap and positioned on the floor of the mesh enclosure (Rat Down). The two animals were separated by 2.5 m. The position of the animals was rotated the following night (Rat Up, Bird Down) and the experiment repeated on consecutive nights. The host animals and position were rotated with each subsequent trial. Mosquitoes entering lard can traps were removed and placed on dry ice for sorting and counting while those remaining in the mesh enclosure were removed by aspiration.

### Data analysis

Confidence intervals surrounding the estimated proportion of *Cx. p. pipiens* selecting a particular host were calculated using the formula 95 % CI = ± 1.96 x (square root *p* (1 – *p*)/n), where *p* = the proportion of *Cx. p. pipiens* attracted to a given animal source, and *n* = the total number of *Cx. p. pipiens* responding (entering the lard can traps) [50]. Before statistical analysis, data were tested with Levene’s test for homogeneity of variance and log (× + 1) transformed. An analysis of variance (ANOVA) was used to determine if host groups or position (up or down) was significant between and within each trial (NDI, DI, OW). After we found a non-significant interaction for group or position, data for each host type were combined to test for differences in the proportion of *Cx. p. pipiens* attracted to the bird or rat by using Pearsons *χ*^2^ analysis for trend. All analyses were performed using IBM SPSS Statistics 21 (IBM, Armonk, NY, USA).

## Results

### Mosquitoes and ovarian dissections

**Fig. 2:**
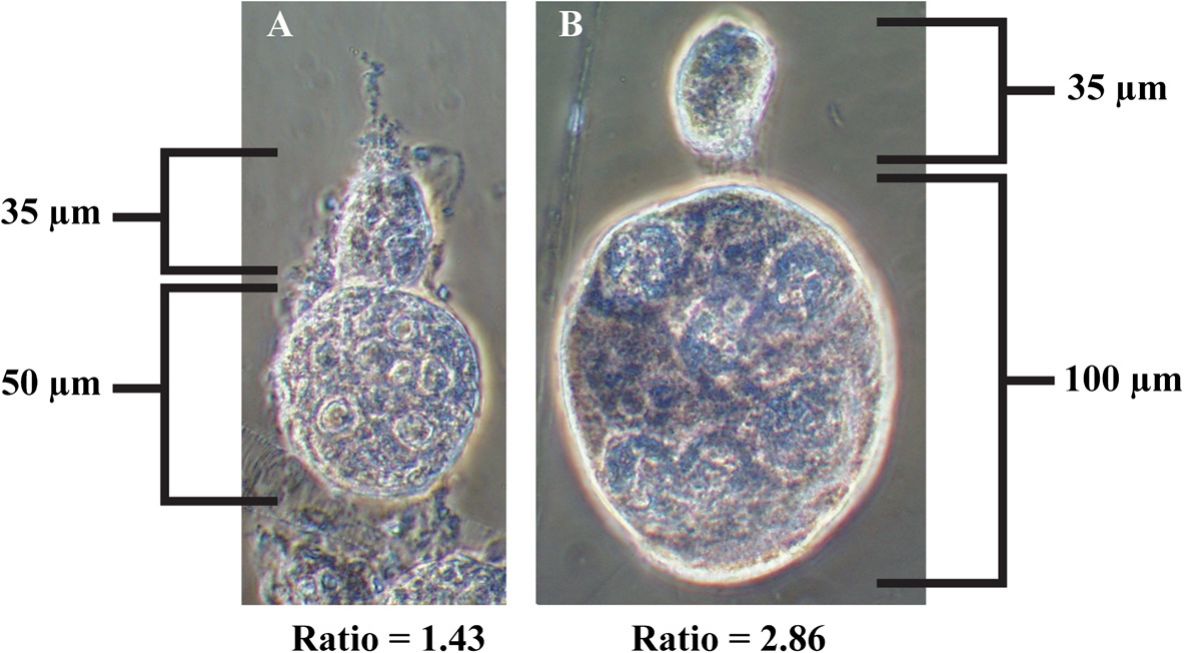
Representative ovarioles from an unengorged *Culex pipiens pipiens*. **a** ovariole from a diapausing mosquito; **b** ovariole from a non-diapause induced mosquito. Ratio values represent the difference in size between the primary and secondary follicles

Non-diapause induced mosquitoes contained primary follicles of 90.13 μm (±0.76 SE), secondary follicles of 31.49 μm (±0.27), and a primary/secondary ratio of 2.90 (±0.03) (Fig. 2). Diapause induced mosquitoes contained primary follicles of 52.38 μm (±0.31), secondary follicles of 38.45 μm (±0.27), and a ratio of 1.37 (±0.01) (Fig. 2). When diapause was terminated in the DI group, their ovarioles had resumed development with primary follicles of 86.98 μm (±0.94), secondary follicles of 31.75 μm (±0.36), and a ratio of 2.76 (±0.03). Field collected OW mosquitoes possessed ovarioles measuring 51.94 μm (±0.28) for primary follicles, 38.79 μm (±0.23) for secondary follicles, and a ratio of 1.34 (±0.01). Terminating diapause in these mosquitoes resumed development of ovarioles and measured 91.20 μm (±0.97) for primary follicles, 34.05 μm (±0.37) for secondary follicles, and a ratio of 2.69 (±0.02).

### Host-choice experiments

**Fig. 3:**
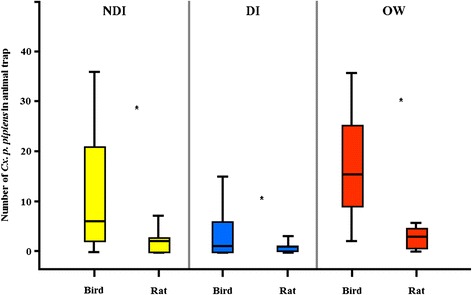
Box plot representing host-choice feeding rates of *Culex pipiens pipiens* under non-diapause induced (NDI), diapause induced (DI), and field overwintering (OW) conditions. Median values are represented by horizontal bar within the boxes and whiskers represent 1.5 SE. Asterisks indicate significant differences between animal hosts (Chi-square tests, *P* < 0.001)

Host seeking *Cx. p. pipiens* were four times more likely to feed on rock doves than rats, regardless of experimental conditions (Table 1). The distribution for the host-choice experiments is represented by the box plots in Fig. 3. Nearly 82 % (n = 694) of *Cx. p. pipiens* preferred the bird versus 18 % (n = 158) for the rat. Under NDI conditions, *Cx. p. pipiens* were significantly (*χ*2 = 377.0; df = 1; *p* < 0.001) more attracted to the bird (79.9 % [75.6–84.1]) than the rat (20.1 [15.9–24.4]) (Table 1). *Culex p. pipiens* exposed to DI conditions were also significantly (*χ*2 = 100.0; df = 1; *p* < 0.001) more attracted to birds (80.0 % [72.2–87.8]) than to rats (20.0 [12.2–27.8]) (Table 1). Overwintering mosquitoes collected from the field and subjected to our host-choice experiments also showed a significant (*χ*2 = 341.0; df = 1; *p* < 0.001) attraction to birds (83.1 % [79.5–86.8]) than to rats (16.9 [13.2–20.5]) (Table 1).

**Table 1:** Host-choice experiment results of Culex pipiens pipiens under non-diapause induced (NDI), diapause induced (DI), and field overwintering (OW) conditions

Condition	Host	No. trials	No. released	No. in all traps (% [95 % CI])	No. in bird trap (% [95 % CI])	No. in rat trap (% [95 % CI])	*χ*2
NDI	Bird up - Rat down	24	959	292 (30.5 [27.5-33.4])	236 (80.8 [76.3-85.3])	56 (19.2 [14.7-23.7])	
	Bird down - Rat up	24	630	51 (8.1 [6.0-10.2])	38 (74.5 [62.6-86.5])	13 (25.5 [13.5-37.5])	
	Total Bird - Rat	48	1,589	343 (21.6 [19.6-23.6])	274 (79.9 [75.6-84.1])	69 (20.1 [15.9-24.4])	*p* < 0.001
DI	Bird up - Rat down	18	349	79 (22.6 [18.3-27.0])	61 (77.2 [68.0-86.5])	18 (22.8 [13.5-32.0])	
	Bird down - Rat up	18	352	21 (6.0 [3.5-8.4])	19 (90.5 [77.9-99.0])	2 (9.5 [0.1-22.1])	
	Total Bird - Rat	36	701	100 (14.3 [11.7-16.9])	80 (80.0 [72.2-87.8])	20 (20.0 [12.2-27.8])	*p* < 0.001
OW	Bird up - Rat down	20	1,331	227 (17.1 [15.0-19.1])	169 (74.5 [68.8-80.1])	58 (25.6 [19.9-31.2])	
	Bird down - Rat up	20	954	182 (19.1 [16.6-21.6])	171 (94.0 [90.5-97.4])	11 (6.0 [2.6-9.5])	
	Total Bird - Rat	40	2,285	409 (17.9 [16.3-19.5])	340 (83.1 [79.5-86.8])	69 (16.9 [13.2-20.5])	*p* < 0.001

## Discussion

Our study shows that when offered a choice, *Cx. p. pipiens* strongly prefers an avian over a mammalian host. Previous laboratory and field studies have also shown distinct ornithophagic patterns for *Cx. p. pipiens* and have even documented behavioral preferences for certain species of birds [14–17, 49, 51]. However, in our experiments the degree of preference did not vary based on physiological status of the mosquitoes (NDI or DI). This indicates that overwintering mosquitoes emerging from hibernaculae in the spring prefer to feed on available wild birds rather than mammals. However, our design provides a measure of preference under laboratory conditions for only a single avian and mammalian species, and does not take into consideration the attractiveness of other hosts from the same class. For example, the host attractiveness of different birds to mosquitoes may vary for each individual species [19, 49]. Additionally, it is also crucial to consider other important variables such as habitat type, vector abundance, genetic ancestry, host density, and host brooding or defensive behavior [17, 52–58].

Previous host feeding studies conducted in the northeastern USA have shown that *Cx. p. pipiens* derives >90 % of their blood meals from birds [14–17], while some studies have reported up to 38 % mammalian feedings [10, 12, 50]. However, mammalian feeding in *Cx. p. pipiens* has been attributed to genetic introgressive hybridization from *Cx. p. pipiens* form molestus populations [10, 12, 18]. But these earlier studies were conducted solely during the mid-summer when *Cx. p. pipiens* populations are abundant, and not during the early season when mosquitoes are emerging from winter diapause. Since molestus populations are incapable of diapause [22, 25], it is improbable that introgression would be occurring in the early season populations. Similar to our experimental overwintering studies, it is expected that overwintering populations undergoing a facultative ovarian diapause are pure populations of *Cx. p. pipiens*. Irrespective, DI and OW mosquitoes in our host-choice experiments which had terminated diapause (to simulate emergence from hibernation in the spring) displayed nearly a 20 % affinity for a mammalian host.

A dilution effect hypothesis has been shown for WNV and nonpasserine diversity before [18, 59, 60], but not during the early season, when the primary enzootic vectors are reinitiating introduction of virus back into host communities. If the primary enzootic amplification vectors feed more indiscriminately on non-competent mammalian hosts, particularly during the most vulnerable period following spring emergence, then this would reduce contact rates away from important avian amplifying hosts and may dampen the intensity of transmission in most host communities. However, during our studies we observed a significant preference for avian hosts by *Cx. p. pipiens*, regardless of the environmental conditions. We again reiterate that our studies were conducted in simulated laboratory conditions and may not reflect the influence of other variables as mentioned above. Further field studies should investigate the host preference of *Cx. p. pipiens* emerging from diapause and evaluate ecological and biological factors that may influence host selection and thus disease dynamics.

## Conclusions

Our findings provide additional information about the innate host preference of *Cx. p. pipiens* emerging from winter diapause in temperate habitats where bridging unfavourable seasons is crucial for disease transmission cycles. For pathogens that utilize the primary vector as a winter reservoir, diapause success not only affects vector population densities, but may also have substantial epidemiological significance. Changes in host preference choices may also contain valuable clues about transmission dynamics and subsequent timely interventions by vector control and public health practitioners. It is also unclear what the impact of global climate change and its effect on diapause dynamics will have on vector populations and pathogen transmission. Future studies, using ecologically relevant temporal, spatial, host, and vector species are required to better assess the role of these variables in disease cycles.

## Abbreviations

Cx. p. pipiens: Culex pipiens pipiens
DI: Diapause induced
L: Linnaeus
NDI: Non-diapause induced
OW: Overwintering
SLE: St. Louis encephalitis virus
WNV: West Nile virus
